# CETP inhibition enhances monocyte activation and bacterial clearance and reduces *streptococcus pneumonia*–associated mortality in mice

**DOI:** 10.1172/jci.insight.173205

**Published:** 2024-04-22

**Authors:** Haoyu Deng, Wan Yi Liang, Le Qi Chen, Tin Ho Yuen, Basak Sahin, Dragoș M. Vasilescu, Mark Trinder, Keith Walley, Patrick C.N. Rensen, John H. Boyd, Liam R. Brunham

**Affiliations:** 1 Department of Medicine, Faculty of Medicine,; 2 Centre for Heart and Lung Innovation, St. Paul’s Hospital,; 3 Department of Microbiology and Immunology, Faculty of Science, and; 4 Department of Experimental Medicine, Faculty of Medicine, University of British Columbia, Vancouver, British Columbia, Canada.; 5 Department of Medicine, Division of Endocrinology, and; 6 Einthoven Laboratory for Experimental Vascular Medicine, Leiden University Medical Center, Leiden, The Netherlands.

**Keywords:** Cardiology, Infectious disease, Bacterial infections, Lipoproteins

## Abstract

Sepsis is a leading cause of mortality worldwide, and pneumonia is the most common cause of sepsis in humans. Low levels of high-density lipoprotein cholesterol (HDL-C) levels are associated with an increased risk of death from sepsis, and increasing levels of HDL-C by inhibition of cholesteryl ester transfer protein (CETP) decreases mortality from intraabdominal polymicrobial sepsis in APOE*3-Leiden.CETP mice. Here, we show that treatment with the CETP inhibitor (CETPi) anacetrapib reduced mortality from *Streptococcus pneumoniae*–induced sepsis in APOE*3-Leiden.CETP and *APOA1.CETP* mice. Mechanistically, CETP inhibition reduced the host proinflammatory response via attenuation of proinflammatory cytokine transcription and release. This effect was dependent on the presence of HDL, leading to attenuation of immune-mediated organ damage. In addition, CETP inhibition promoted monocyte activation in the blood prior to the onset of sepsis, resulting in accelerated macrophage recruitment to the lung and liver. In vitro experiments demonstrated that CETP inhibition significantly promoted the activation of proinflammatory signaling in peripheral blood mononuclear cells and THP1 cells in the absence of HDL; this may represent a mechanism responsible for improved bacterial clearance during sepsis. These findings provide evidence that CETP inhibition represents a potential approach to reduce mortality from pneumosepsis.

## Abstract 2

The cholesteryl ester transfer protein inhibitor anacetrapib reduces mortality from <em>Streptococcus pneumoniae</em>-induced sepsis in mice by enhancing monocyte activation, improving bacterial clearance, and reducing organ injury.

## Graphical abstract

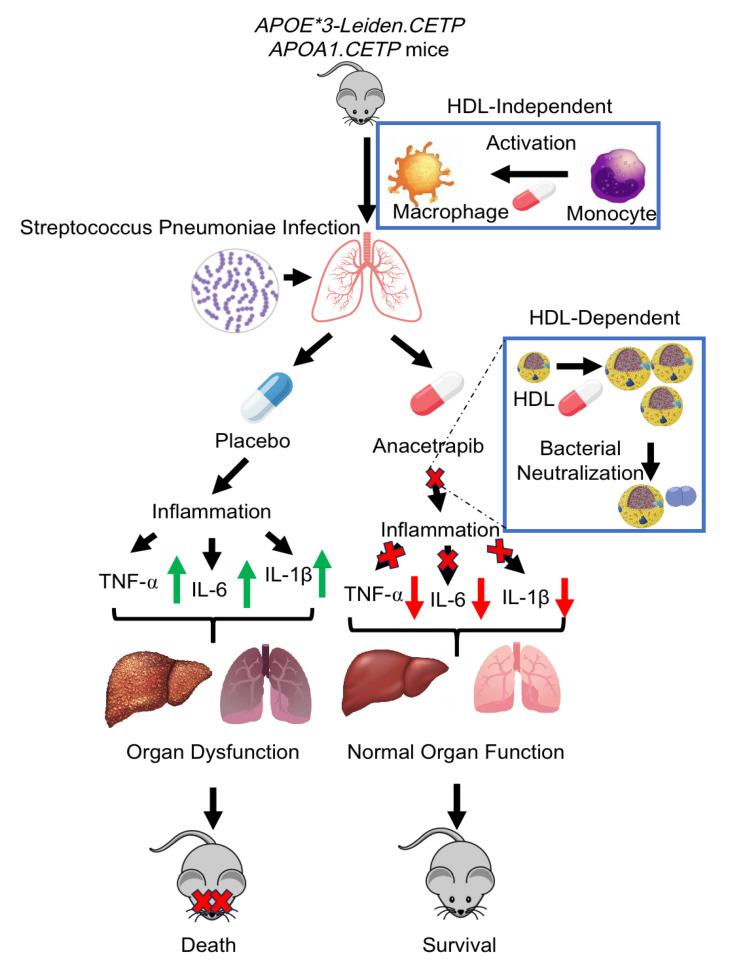

## Introduction

Sepsis is defined as life-threatening organ dysfunction caused by a dysregulated host response to infection and is responsible for substantial morbidity, mortality, and healthcare expenditure worldwide (1, 2). The pathophysiology of sepsis centers on the release of inflammatory mediators in response to invading microorganisms (3). Current treatment approaches involve fluid resuscitation and early administration of antibiotics. However, even with aggressive treatment, the mortality of septic shock remains 40%–80% (4), emphasizing the urgent need for new sepsis therapies.

An important component of the pathophysiology of sepsis is the release of pathogen-associated lipids (PALs), such as LPS from Gram-negative bacteria and lipoteichoic acid (LTA) from Gram-positive bacteria, during bacterial proliferation and after bacterial death and phagocytosis in the liver and spleen, leading to exaggerated activation of the immune system (5). The central role of PALs in sepsis is underscored by the observation that total systemic exposure to circulating PALs is directly correlated to the degree of organ failure (6). PALs can be sequestered and neutralized by lipoproteins such as high-density lipoprotein (HDL) and low-density lipoprotein (LDL) (7). In comparison with LDL, HDL has the greatest affinity for binding to PALs (8). Plasma levels of HDL decrease during sepsis, and a lower level of HDL-cholesterol (HDL-C) is associated with a greater risk of organ dysfunction and death (9, 10). Mendelian randomization studies implicate a causal role for HDL, but not LDL, in survival from sepsis (11). HDL particles have many properties that may be relevant to improving survival from sepsis, including their role in regulating host inflammation and their ability to inhibit inflammation by suppressing chemokine expression (12–14). More recently, isolated macrophages treated with HDL have been shown to enhance bacterial phagocytosis, and mice with higher reconstituted HDL levels have more effective clearance of bacterial infection (15–17).

The cholesteryl ester transfer protein (CETP) is a key determinant of HDL-C levels (18). Gain-of-function genetic variants in CETP are associated with a large decline in HDL-C during sepsis and with increased risk of death (19). Inhibition of CETP in APOE*3-Leiden.CETP mice, a well-established mouse model of human-like lipoprotein metabolism, after the onset of intraabdominal sepsis prevents the decline in HDL-C levels and improves survival (9). However, whether CETP inhibition is beneficial in more clinically relevant models of sepsis, such as pneumonia, is unknown. In addition, the mechanisms by which CETP inhibition and HDL improve survival from sepsis remain incompletely understood. The goals of this study were to investigate the effects and cellular mechanisms of CETP inhibition in a mouse model of pneumonia and in vitro model systems.

## Results

### CETP inhibition improves Streptococcus pneumoniae–induced sepsis mortality in APOE*3-Leiden.CETP mice.

**Figure 1:**
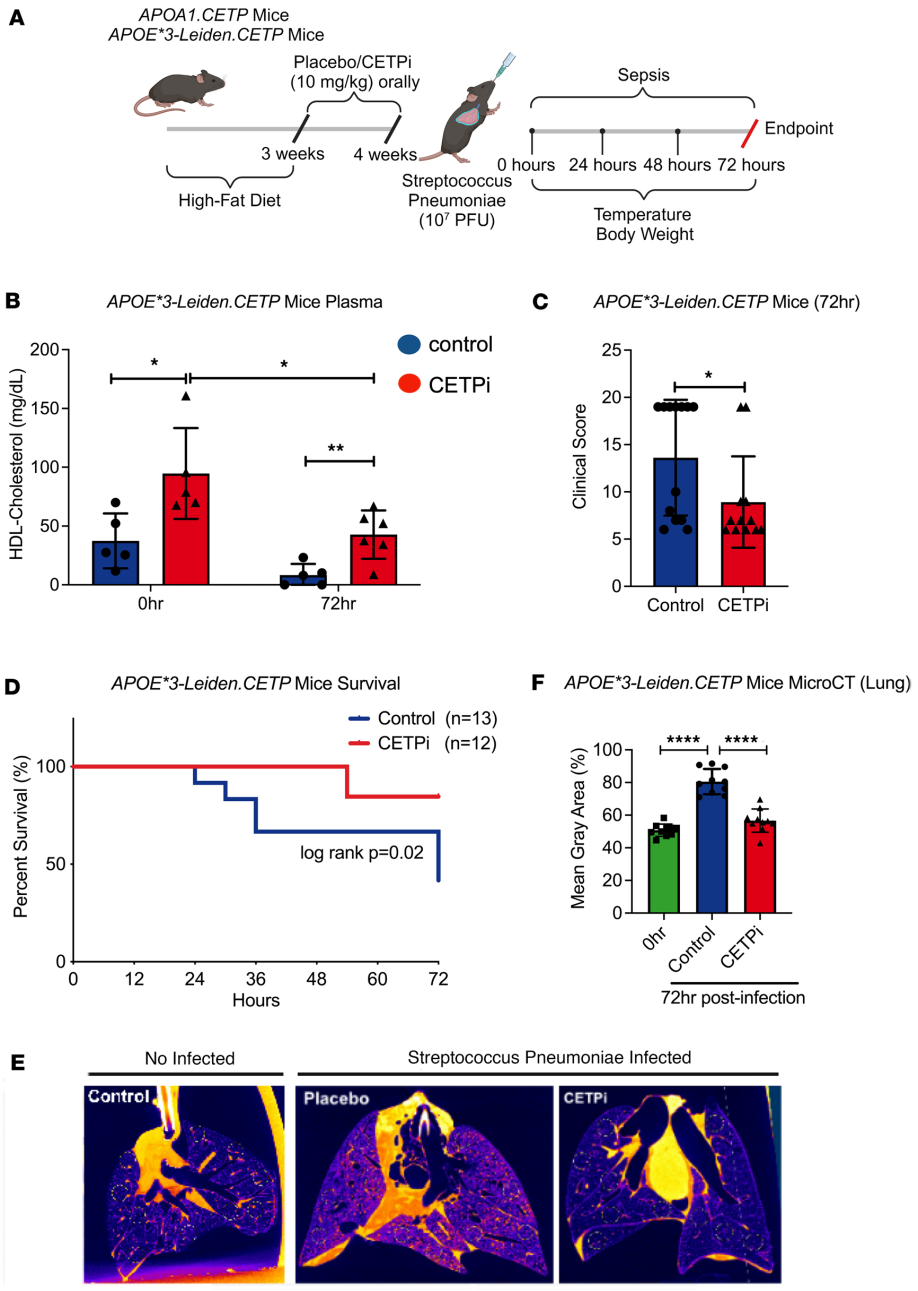
CETPi significantly improves *Streptococcus pneumoniae–*induced sepsis mortality in APOE*3-Leiden.CETP mouse model. (**A**) Schematic of the *Streptococcus pneumoniae*–induced sepsis model used in the study. (**B**) Plasma HDL-C levels in samples obtained 0 (CETPi [*n* = 5] versus Control [*n* = 5]): mean ± SD, 94.7 ± 38.6 versus 37.4 ± 23.3 mg/dL, unpaired *t* test, *P* = 0.02 with Bonferroni correction and 72 hours (mean ± SD, 42.8 ± 20.6 [CETPi, *n* = 6] versus 8.3 ± 9.5 [Control, *n* = 5] mg/dL, unpaired *t* test with Bonferroni correction, *P* = 0.007) after *S*. *pneumoniae* infection from female APOE*3-Leiden.CETP mice treated with control or CETPi. (**C**) Murine Clinical Assessment Score for sepsis for female APOE*3-Leiden.CETP mice at 72 hours after infection (mean ± SD, 8.9 ± 4.8 [CETPi, *n* = 12] versus 13.6 ± 6.1 [Control, *n* = 13], unpaired *t* test, *P* = 0.04). (**D**) Kaplan-Meier plot of female APOE*3-Leiden.CETP mice survival rate (72-hour survival rate: 83.3% [CETPi, *n* = 12] versus 38.5% [Control, *n* = 13], log-rank: *P* = 0.02). (**E** and **F**) Representative micro-CT lung figures of female noninfected APOE*3-Leiden.CETP mice and mice treated with control or CETPi at 72 hours after infection. Quantification of inflammation was determined by random sampling of 10 spots on each pair of lungs and measuring gray matter density using ImageJ (mean ± SD, 56.6 ± 7.1% [CETPi, *n* = 10] versus 80.5 ± 7.7 [Control, *n* = 10], 1-way ANOVA, *P* < 0.0001). Data are shown as mean ± SD. *P < 0.05, **P < 0.01, ****P < 0.0001. ApoE, apolipoprotein E; CETP, cholesteryl ester transfer protein; HDL, high-density lipoprotein cholesterol.

Previously, we demonstrated a protective role of CETP inhibition in an intraabdominal model of sepsis (9). In humans, pneumonia is the leading cause of sepsis (20), and we therefore tested the effect of CETP inhibition in a mouse model of lower respiratory tract infection. We used APOE*3-Leiden.CETP mice because of their humanized lipid profile and large HDL-raising effect from CETP inhibition (21). Female APOE*3-Leiden.CETP mice were fed with a cholesterol-containing diet for 3 weeks, followed by the treatment with CETP inhibitor (CETPi), or vehicle-only control, orally for 1 week and subsequently underwent intratracheal inoculation with *S*. *pneumoniae* (Figure 1A). CETP activity levels were reduced at 72 hours after infection in mice treated with CETPi (Supplemental Figure 1A; supplemental material available online with this article; https://doi.org/10.1172/jci.insight.173205DS1). CETPi treatment resulted in increased HDL-C levels compared with control baseline (t = 0 hours) (Figure 1B). HDL-C levels dropped in both groups at 72 hours but remained higher in mice treated with CETPi (Figure 1B). The percent reduction in HDL-C was less in CETPi-treated mice versus control mice (55% versus 78%) (Figure 1B). We did not observe significant changes in LDL-C and total cholesterol between 2 groups (Supplemental Figure 1, B and C).

Mice treated with CETPi maintained their body weight and temperature as compared with those with control treatment (Supplemental Figure 1, D and E). CETPi treatment resulted in a significantly lower murine clinical assessment score compared with control, indicating less severe sepsis (Figure 1C). This improvement was reflected in significantly lower mortality of APOE*3-Leiden.CETP mice treated with CETPi versus control (Figure 1D). Micro-CT of air-inflated lungs obtained after euthanization of the mice revealed extensive inflammatory changes in control-treated mice, with substantially less extensive radiographic changes (mean grey area) in mice treated with CETPi (Figure 1, E and F).

### CETP inhibition improves S. pneumoniae–induced sepsis mortality in APOA1.CETP mice.

**Figure 2:**
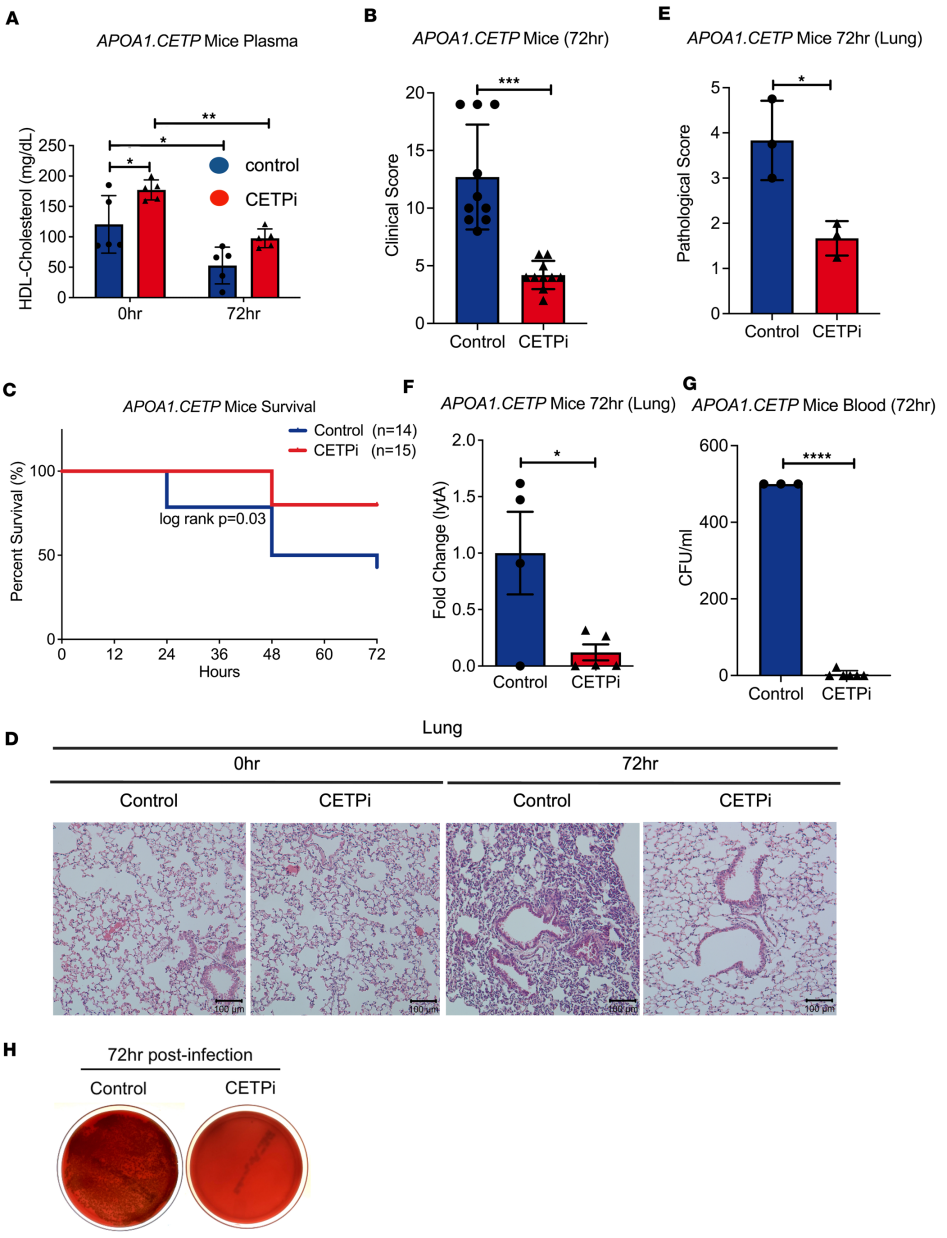
CETPi significantly improves *Streptococcus pneumoniae–*induced sepsis mortality in *APOA1.CETP* mice model. (**A**) Plasma HDL-C levels in samples obtained 0 hours (mean ± SD, 177.2 ± 16.5 [CETPi, *n* = 5] versus 120.5 ± 47.3 [Control, *n* = 5] mg/dL, 2-way ANOVA, *P* = 0.0412) after infection from female *APOA1.CETP* mice treated with control or CETPi. (**B**) Murine Clinical Assessment Score for sepsis for female *APOA1.CETP* mice at 72 hours after infection score (mean ± SD, 12.7 ± 4.5 [CETPi, *n* = 10] versus 4.2 ± 1.2 [Control, *n* = 10], unpaired *t* test, *P* = 0.00002). (**C**) Kaplan-Meier plot of female *APOA1.CETP* mice survival rate (72-hour survival rate: 80.0% [CETPi, *n* = 15] versus 42.8% [Control, *n* = 14], log-rank: *P* = 0.03). (**D**) Representative H&E staining figures of lungs harvested at 0 hours and 72 hours after infection from female APOE*3-Leiden.CETP mice treated with control or CETPi. (**E**) Pathological score was determined based on intensity of injury and inflammation (mean ± SD, 3.8 ± 0.9 [Control, *n* = 3] versus 1.7 ± 0.4 [CETPi, *n* = 3], unpaired *t* test, *P* = 0.02). Lung injury scoring system: 0, normal lung; 1, neutrophils in the alveolar space; 2, neutrophils in the interstitial space; 3,hyaline membranes; 4, proteinaceous debris filling the airspaces; 5, alveolar septal thickening. (**F**) Relative mRNA expression of *S*. *pneumoniae* gene *lytA* in lung homogenate at 72 hours after infection of female *APOA1.CETP* mice treated with control or CETPi (mean ± SD, 0.12 ± 0.16 [CETPi, *n* = 5] versus 1.0 ± 0.73 [Control, *n* = 4] relative gene expression, unpaired *t* test, *P* = 0.01). (**G** and **H**) Representative bacterial growth on blood agar plates from plasma obtained 72 hours after infection from female *APOA1.CETP* mice treated with control or CETPi (mean ± SD, 3.7 ± 9.0 [CETPi, *n* = 3] versus 500.0 ± 0.0 [Control, *n* = 6] CFU/mL, unpaired *t* test, *P* = 4.5 × 10^–12^). CFU > 500 units were determined as 500 on graph. Data are shown as mean ± SD. *P < 0.05, **P < 0.01, ***P < 0.001, ****P < 0.0001. ApoA1, apolipoprotein A-1; CETP, cholesteryl ester transfer protein; CFU, colony-forming unit; HDL, high-density lipoprotein cholesterol; LytA, autolysin.

To further evaluate the robustness of these findings, we used the same experimental design in *APOA1.CETP* mice (Figure 1A). We observed similar trends in plasma HDL-C levels with statistically significant increases in CETPi- versus control-treated mice observed at time 0 hours and 72 hours after infection (Figure 2A). The percent reduction in HDL-C was less in CETPi-treated mice versus control mice (45% versus 56%) (Figure 2A). We observed similar and statistically significant improvements in murine clinical assessment (Figure 2B) and mortality in mice treated with CETPi in this model (Figure 2C). Likewise, mice treated with CETPi had preservation of body weight and temperature (Supplemental Figure 2, A and B). H&E staining of lung tissue demonstrated that, at 72 hours after infection, control-treated mice had more expansive, suppurative to necrotizing bronchopneumonia with, predominantly, neutrophil infiltration within bronchial lumina and alveoli (Figure 2D). Pathological scores at 72 hours were significantly higher than those observed in CETPi-treated mice (Figure 2E). To quantify the burden of *S*. *pneumoniae* infection, we determined the mRNA expression of *lytA*, the major autolysin-encoding gene for *S*. *pneumoniae*. We found that *lytA* expression was significantly lower in CETPi-treated mice as compared with control, indicating reduced pneumococcal infection (Figure 2F). This result was confirmed by the lower presence of bacteria from blood collected 72 hours after infection in CETPi-treated versus control mice (Figure 2, G and H). Taken together, these results establish that CETP inhibition led to significantly improved survival and less extensive pneumonia in 2 independent mouse models.

### CETPi treatment decreases proinflammatory cytokines in bronchoalveolar lavage fluid, plasma, and liver.

**Figure 3:**
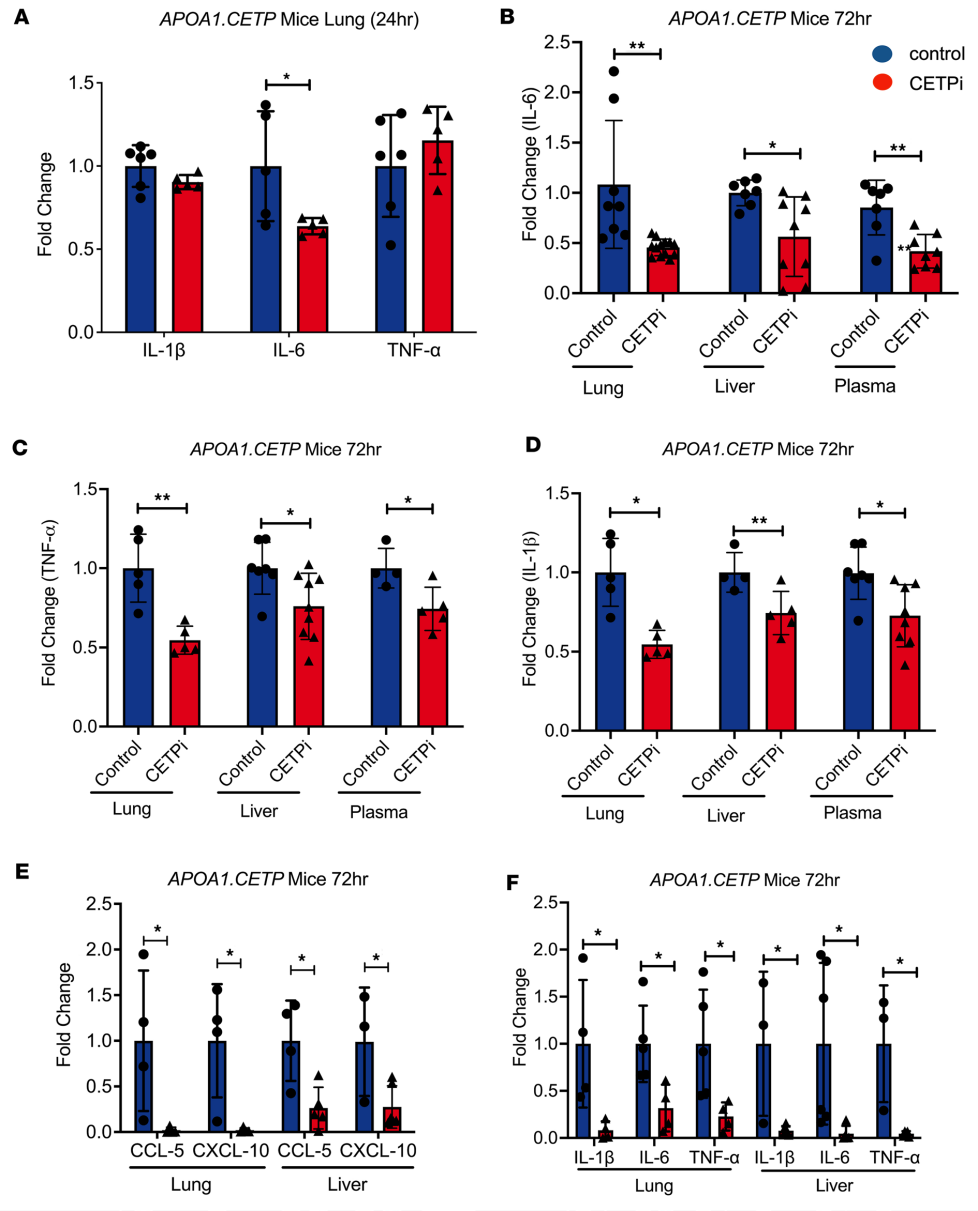
CETPi significantly decreases secretion of proinflammatory cytokines in *S. pneumoniae*–infected mice. (**A**) Levels of secreted IL-6, IL-1β, and TNF-α in lung samples obtained 24 hours after *S. pneumoniae* infection from female APOA1.CETP mice treated with CETPi or control (IL-6: mean ± SD, 0.64 ± 0.05 versus 1.0 ± 0.3, unpaired *t* test, *P* = 0.04). (**B**–**D**) Levels of secreted IL-6, IL-1β, and TNF-α in lung (IL-6 lung, mean ± SD, 1.0 ± 0.6 versus 0.42 ± 0.08, unpaired *t* test, *P* = 0.002), plasma (IL-6, mean ± SD, 0.49 ± 0.2 versus 1.0 ± 0.3, unpaired *t* test, *P* = 0.002), and liver (IL-6, mean ± SD, 0.56 ± 0.4 versus 1.0 ± 0.1 unpaired *t* test, *P* = 0.01). IL-1β, lung: mean ± SD, 0.63 ± 0.10 versus 1.0 ± 0.1, unpaired *t* test, *P* = 0.003; liver: mean ± SD, 0.7 ± 0.2 versus 1.0 ± 0.3, unpaired *t* test, *P* = 0.03; plasma: mean ± SD, 0.75 ± 0.20 versus 1.0 ± 0.18, unpaired *t* test, *P* = 0.04. TNF-α: lung: mean ± SD, 0.55 ± 0.09 versus 1.0 ± 0.2, unpaired *t* test, *P* = 0.002; liver: mean ± SD, 0.74 ± 0.13 versus 1.0 ± 0.1, unpaired *t* test, *P* = 0.02; plasma: mean ± SD, 0.76 ± 0.21 versus 1.0 ± 0.2, unpaired *t* test, *P* = 0.02. (**E**) Transcriptional levels of proinflammatory cytokines in lung and liver 72 hours after infection from female APOA1.CETP mice treated with control or CETPi (Lung: IL-6: mean ± SD, 1.0 ± 0.4 versus 0.32 ± 0.25, unpaired *t* test, *P* = 0.02; IL-1β: mean ± SD, 1.0 ± 0.7 versus 0.08 ± 0.09, unpaired *t* test, *P* = 0.04; TNF-α: mean ± SD, 1.00 ± 0.06 versus 0.23 ± 0.15, unpaired *t* test, *P* = 0.04. Liver: IL-6: mean ± SD, 1.0 ± 0.9 versus 0.04 ± 0.03, unpaired *t* test, *P* = 0.04; IL-1β: mean ± SD, 1.0 ± 0.8 versus 0.08 ± 0.05, unpaired *t* test, *P* = 0.02; TNF-α: mean ± SD, 1.0 ± 0.6 versus 0.05 ± 0.08, unpaired *t* test, *P* = 0.01). (**F**) Transcriptional levels of chemokines in lung and liver 72 hours after infection from female APOA1.CETP mice treated with control or CETPi (Lung: CXCL-10: mean ± SD, 1.0 ± 0.8 versus 0.02 ± 0.03, unpaired *t* test, *P* = 0.04; CCL5: mean ± SD, 1.0 ± 0.6 versus 0.02 ± 0.02, unpaired *t* test, *P* = 0.02. Liver: CXCL-10: mean ± SD, 1.0 ± 0.6 versus 0.28 ± 0.23, unpaired *t* test, *P* = 0.03; CCL5: mean ± SD, 1.0 ± 0.4 versus 0.26 ± 0.23, unpaired *t* test, *P* = 0.01). Data displayed as mean ± SD. *P < 0.05, **P < 0.01, ***P < 0.001. ApoA1, apolipoprotein A-1; CCL-5, chemokine ligand 5; CETP, cholesteryl ester transfer protein; CXCL-10, C-X-C motif chemokine ligand 10.

We next sought to identify the mechanisms by which CETP inhibition results in improved survival from pneumosepsis. We hypothesized that CETPi would act to reduce the host inflammatory response, leading to less severe inflammation and organ damage. We examined the production of proinflammatory cytokines in *APOA1.CETP* mice using ELISA. IL-1β, IL-6, and TNF-α are all potent proinflammatory cytokines that contribute heavily to organ dysfunction from sepsis (22–24). First, we determined the quantity of production for IL-1β, IL-6, and TNF-α in the lung as the primary site of infection. We found that levels of IL-6 at 24 hours after infection was significantly lower in the lungs of mice treated with CETPi compared with control, whereas levels of IL-1β and TNF-α were unchanged (Figure 3A). CETPi treatment was also associated with decreased IL-6 levels in bronchoalveolar lavage fluid (BAL) at 72 hours after infection (Figure 3B). We also found that IL-6 levels at 72 hours after infection were significantly lower in the plasma of mice treated with CETPi, as compared with those with control (Figure 3B). Since the liver is responsible for proinflammatory cytokine production and bacterial clearance during sepsis (25, 26), we also examined IL-6 levels in liver homogenates. We found that CETPi led to significantly lowered IL-6 levels at 72 hours after infection (Figure 3B). Although IL-1β and TNF-α levels were not significantly regulated by CETP inhibition in lung or BAL at 24 hours, we found that, at 72 hours, IL-1β and TNF-α levels were significantly lower in the lung or BAL, plasma, and liver of CETPi-treated mice at 72 hours (Figure 3, C and D). To determine if CETP inhibition regulates proinflammatory cytokine production at the transcriptional level, we examined the mRNA levels of IL-6, IL-1β, and TNF-α using quantitative PCR (qPCR) at 72 hours after infection. This revealed that the mRNA levels of IL-6, IL-1β, and TNF-α were all significantly decreased in both the lung and liver of CETPi-treated mice versus control mice (Figure 3E). We also found that the transcriptional levels of proinflammatory chemokines CXCL-10 and CCL5 were significantly reduced with CETPi treatment compared with control (Figure 3F). Overall, these results show that CETP inhibition leads to a reduction in the host proinflammatory response via attenuation of proinflammatory cytokine release and transcription.

We next asked whether the observed attenuation of host inflammation CETPi treatment is attributable to increased HDL or to other effects of CETPi. To address this, we treated human monocyte THP1 cells with serum from CETPi-treated mice. We found that serum from CETPi-treated mice significantly reduced the protein expression of COX-2 and the release of and IL-1β in LPS-stimulated THP1 cells (Supplemental Figure 3, A–C). In contrast, treating THP1 cells with CETPi in the absence of serum from CETPi-treated mice did not reduce LPS-stimulated IL-1β release (Supplemental Figure 3D), suggesting that the effects of CETPi on reducing host inflammation are dependent on the presence of HDL.

### CETP inhibition enhances macrophage infiltration to the site of infection and monocyte activation at different stages of sepsis.

**Figure 4:**
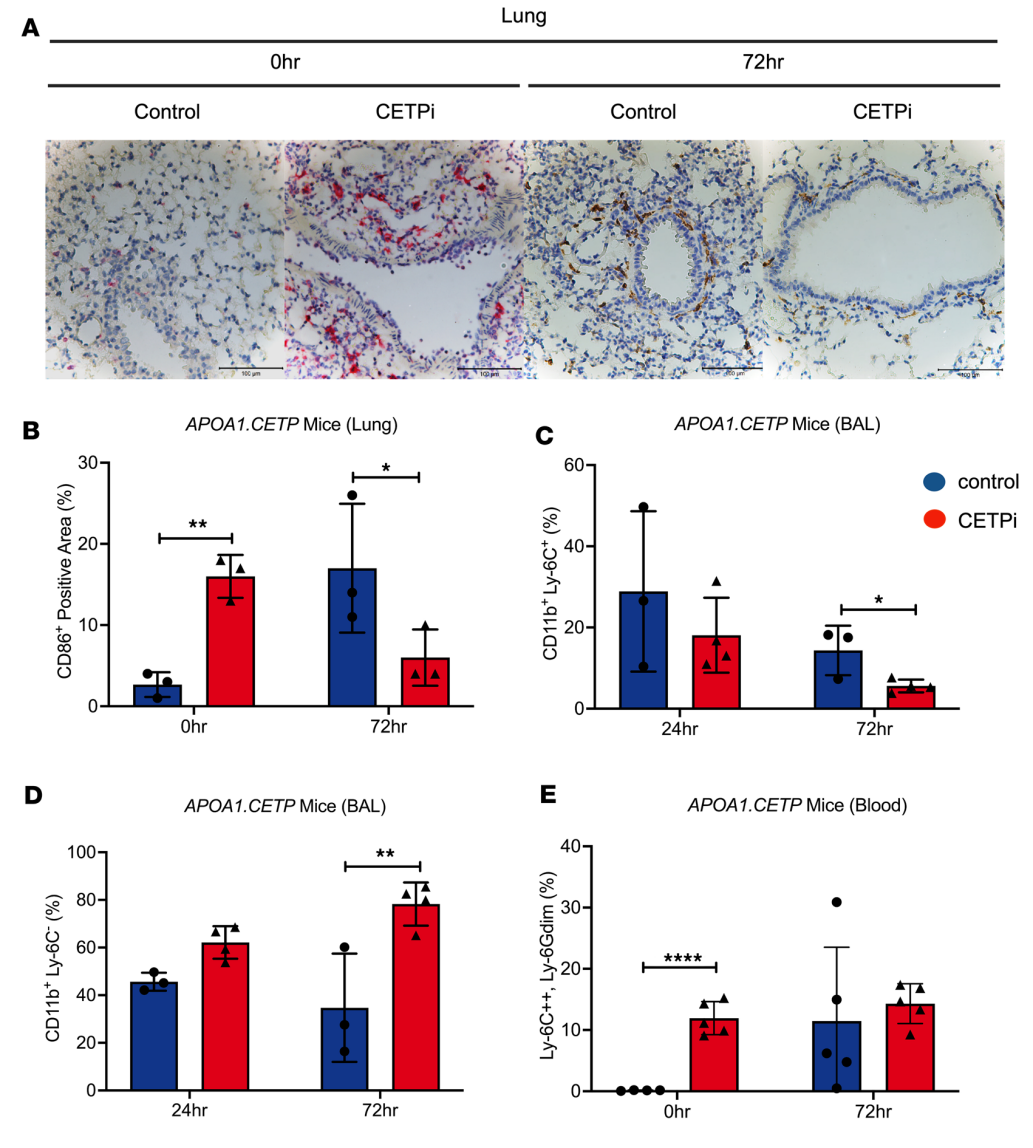
CETPi increases macrophage infiltration into site of infection and macrophage activation while maintaining tissue homeostasis at different stages of sepsis. (**A** and **B**) Representative staining figures of M1 macrophages by marker CD86 in lung obtained 0 and 72 hours after infection by *S*. *pneumoniae* from female *APOA1.CETP* mice treated with control or CETPi. Quantification was determined by random sampling spots on lungs of 3 different mice in each treatment and by measuring stained areas using ImageJ (H&E 0 hours, mean ± SD, 16.0 ± 2.6 [CETPi, *n* = 3] versus 2.7 ± 1.5 [Control, *n* = 3], unpaired *t* test, *P* = 0.002) (IHC 72 hours, mean ± SD, 6.0 ± 3.5 [CETPi, *n* = 3] versus 17.0 ± 7.9% [Control, *n* = 3], unpaired *t* test, *P* = 0.02). (**C** and **D**) Proportion of infiltrating inflammatory macrophages (*CD11b^+^Ly6c^+^*) and tissue repairing and inflammation resolving macrophages (*CD11b^+^Ly6c^–^*) in BAL samples obtained 24 and 72 hours after infection from female *APOA1.CETP* mice treated with control or CETPi (*CD11b^+^Ly6c^+^* 72 hours, mean ± SD, 5.6 ± 1.8 [CETPi, *n* = 4] versus 14.3 ± 6.1% [Control, *n* = 3], unpaired *t* test with Bonferroni correction, *P* = 0.037) (*CD11b^+^ Ly6c^–^* 24 hours, mean ± SD, 62.1 ± 6.8 [CETPi, *n* = 4] versus 45.6 ± 3.8 [Control, *n* = 3], unpaired *t* test with Bonferroni correction, *P* = 0.013; *CD11b^+^ Ly6c^–^* 72 hours, mean ± SD, 78.3 ± 9.1 [CETPi, *n* = 4] versus 34.7 ± 22.7 [Control, *n* = 3], unpaired *t* test with Bonferroni correction, *P* = 0.016). (**E**) Proportion of migrating monocytes (*Ly6C*^++^*Ly6G^DIM^*) in blood samples obtained 0 and 72 hours after infection from female *APOA1.CETP* mice treated with control or CETPi (mean ± SD, 12.0 ± 2.7 [CETPi, *n* = 5] versus 0.14 ± 0.06 [Control, *n* = 4], unpaired *t* test, *P* = 0.00005). Data are shown as mean ± SD. *P < 0.05, **P < 0.01, ****P < 0.0001. ApoA1, apolipoprotein A-1; CETP, cholesteryl ester transfer protein; CD86, cluster of differentiation 86; CD11b, integrin α M; Ly6-C, lymphocyte antigen 6 locus C; Ly6-G, lymphocyte antigen 6 locus G.

Since CETPi decreased proinflammatory cytokine production upon *S*. *pneumoniae* challenge, we next used IHC and flow cytometry to investigate the effect of CETP inhibition on the activation of macrophages, the primary cell type involved in the release of proinflammatory cytokines in the innate immune response (27). Proinflammatory Ly6C^hi^ monocytes are derived in the bone marrow and, in the absence of infection, differentiate into patrolling Ly6C^lo^ monocytes that survey the vasculature and take part in tissue repair and healing. During infection, proinflammatory Ly6C^hi^ monocytes are recruited into the site of inflammation, where they differentiate into inflammatory macrophages that produce high levels of inflammatory cytokines such as IL-6, TNF-α, IL-1β. We performed IHC staining of lungs at 0 hours to investigate the effect of CETPi on macrophage activation prior to bacterial challenge. We observed that CETPi-treated *APOA1.CETP* mice had higher levels of proinflammatory macrophages in their lungs at 0 hours, suggesting that CETP inhibition primed the influx of proinflammatory macrophages into the organs even in the absence of *S*. *pneumoniae* (Figure 4, A and B). We then focused on the host response to *S*. *pneumoniae* by investigating the percentage of infiltrating macrophages in BAL. In normal lungs, tissue-resident *CD11b^–^* alveolar macrophages replenish the macrophage pool of lungs. However, during injury, inflammation, or infection, circulating *CD11b^+^* monocytes are recruited into the lung, where they differentiate into monocyte-derived alveolar macrophages and play a crucial role in initiating and amplifying the inflammatory response (28). At 72 hours after infection, the percentage of *CD11b^+^Ly6C^hi^* macrophages in the BAL were significantly lower in the CETPi-treated group compared with the control group (Figure 4C). This finding was confirmed by IHC staining on lung tissue at 72 hours after infection, and this showed fewer CD86^+^ macrophages in CETPi-treated mice (Figure 4, A and B). This suggests that CETP inhibition may prevent excessive inflammatory response–induced organ damage or may be a result of lower bacterial burden in the later stages of sepsis.

Additionally, we found that the percentage of *CD11b^+^Ly6C^lo^* macrophages, a subtype of macrophages that promote tissue repair and the resolution of inflammation, was significantly higher in the CETPi-treated group compared with the control group at both 24 hours and 72 hours (Figure 4D). To further examine the effect of CETPi on differential activation of monocytes, we measured the expression of Ly6C in the circulating monocytes in blood. We identified a population of monocytes that have very high expression of Ly6C (*Ly6C*^++^) and low expression of Ly6G (*Ly6G^DIM^*); Ly6G is a marker of myeloid precursors in bone marrow that are lost on monocytes as migrate out of the bone marrow (29, 30). We observed a significant increase in the proportion of *Ly6C*^++^*Ly6G^DIM^* monocytes in the CETPi-treated group compared with control-treated mice at 0 hours (Figure 4E). We observed a similar effect in APOE*3-Leiden.CETP mice, with increased *Ly6C*^++^*SS^hi^* cells in blood from CETPi-treated mice at 0 hours (Supplemental Figure 4A). Collectively, these results suggest that CETP inhibition promotes proinflammatory macrophage infiltration into the lung and macrophage activation to support the host immune response at the early phase of sepsis.

### CETPi increases caspase-1 and COX-2 protein expression in human and murine cells.

**Figure 5:**
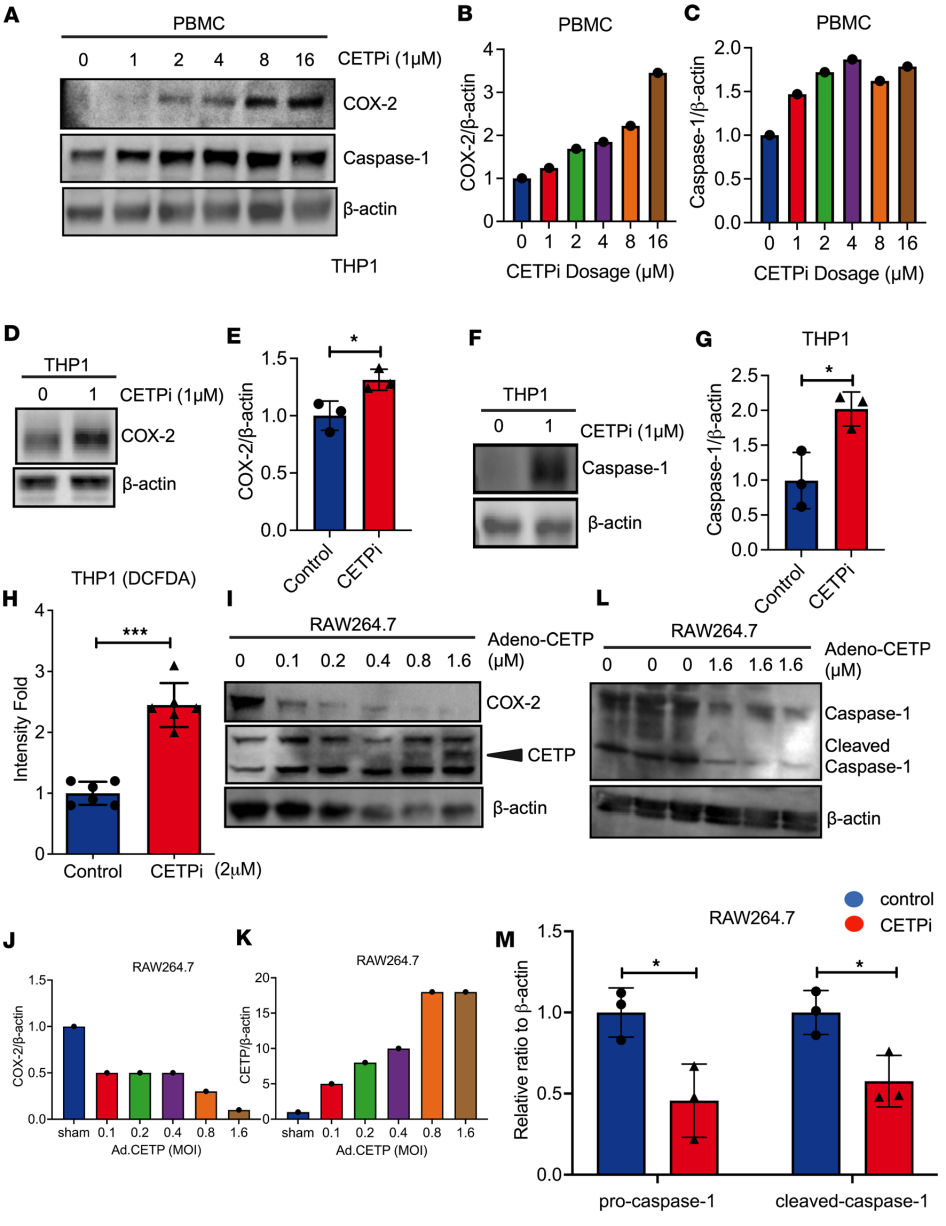
CETPi decreases caspase-1 and COX-2 protein expression in human and mouse cells. (**A**–**C**) COX-2 and Caspase-1 expression in PBMCs treated with increasing doses of CETPi. (**D**–**E**) COX-2 expression in THP1 cells treated with control or CETPi (1 μM) (mean ± SD, 1.00 ± 0.42 [Control, *n* = 3] versus 1.31 ± 0.38 [CETPi, *n* = 3], unpaired *t* test, *P* = 0.03). (**F**–**G**) Caspase-1 in THP1 cells treated with control or CETPi (1 μM) (mean ± SD, 1.00 ± 0.19 [Control, *n* = 3] versus 2.02 ± 0.11 [CETPi, *n* = 3], unpaired *t* test, *P* = 0.02). (**H**) Expression of ROS in THP1 cells treated with control or CETPi (2 μM), (mean ± SD, 2.45 ± 0.36 [CETPi, *n* = 6] versus 1.00 ± 0.19 [Control, *n* = 6], unpaired *t* test, *P* = 0.00005). (**I**–**K**) Expression of COX-2 and CETP in RAW264.7 cells after adenovirus transfection induced-CETP overexpression in increasing MOIs. (**L**–**M**) Expression of pro–caspase-1 and cleaved caspase-1 in RAW 264.7 cells after adenovirus transfection–induced CETP overexpression at MOI = 1.6. Pro–caspase-1: mean ± SD, 1.00 ± 0.15 (Control, *n* = 3) versus 0.45 ± 0.22 (CETP overexpress, *n* = 3), unpaired *t* test, *P* = 0.02; cleaved caspase-1: mean ± SD, 1.00 ± 0.14 (Control, *n* = 3) versus 0.58 ± 0.16 (CETP overexpress, *n* = 3), unpaired *t* test, *P* = 0.03. Data displayed as mean ± SD. *P < 0.05, **P < 0.01, ***P < 0.001. Caspase-1, caspase-1/IL-1 converting enzyme; COX-2, prostaglandin-endoperoxide synthase 2; MOI, multiplicity of infection; PBMC, human peripheral blood mononuclear cell; RAW, murine macrophage cell; ROS, reactive oxygen species; THP1, human peripheral blood monocyte.

The observation that anacetrapib enhanced the activation of proinflammatory monocytes prior to bacterial infection suggests an immunomodulatory effect of CETPi, even in the absence of bacterial infection. To further examine whether CETPi can directly regulate monocyte activation, independently of changes in HDL levels, we next tested the effects of CETPi on monocyte activation in vitro. We examined the protein expression levels of cyclo-oxygenase 2 (COX-2) and caspase-1, 2 markers of monocyte activation, in human peripheral blood mononuclear cells (PBMCs) and human peripheral blood monocytes cell line (THP1) by Western blotting (31, 32). We observed that COX-2 expression in PBMCs increased in a dose-dependent manner with CETPi treatment (Figure 5, A and B). The expression of caspase-1 in PBMC was enhanced in a dose-dependent manner with an increase in CETPi concentration to 4 μM. Furthermore, the expression of caspase-1 remained at a high level when the CETPi concentration was further increased (Figure 5, A and C). We observed the same trends in the THP1 cell line (Figure 5, D–G). Since reactive oxygen species (ROS) are essential for macrophages to eliminate invading bacteria, we examined ROS expression in THP1 cells in response to CETPi. ROS expression significantly increased in THP1 cells treated with CETPi in comparison with those treated with vehicle control (Figure 5H). To determine if the increase in COX-2 and caspase-1 is directly attributable to the effects of CETP, we tested the effect of CETP overexpression on COX-2 inhibition in mouse RAW 264.7 macrophages that lack endogenous CETP. Overexpression of CETP led to a reduction in COX-2 (Figure 5, I–K) and caspase-1 (Figure 5, L and M) expression. In the absence of CETP overexpression, CETPi treatment had no effect on the gene expression of *IL-1β*, *IL-6*, *TNF-α*, *COX-2*, or *CASP-1* of RAW 264.7 cells, indicating that the effects of CETPi are dependent on the presence of CETP (Supplemental Figure 5A). These findings suggest that CETPi has direct effects on monocyte activation and that inhibition of CETP leads to upregulated monocyte activation, which may contribute to the enhanced bacterial clearance and improved survival during sepsis observed in mice treated with anacetrapib. To further determine if the observed effects of anacetrapib on macrophage activation are unique to anacetrapib, or represent a broader class effect of CETPi, we tested the effects of evacetrapib, another CETPi, on the expression of *CASP-1* and *COX-2* in THP1 cells. We found that evacetrapib significantly upregulated the gene expression of *CASP-1* and *COX-2* (Supplemental Figure 5, B and C), suggesting the immunomodulatory effects of CETP inhibition represent a class-effect rather than being specific to anacetrapib.

### CETPi treatment after infection significantly reduces S. pneumoniae–induced sepsis mortality.

**Figure 6:**
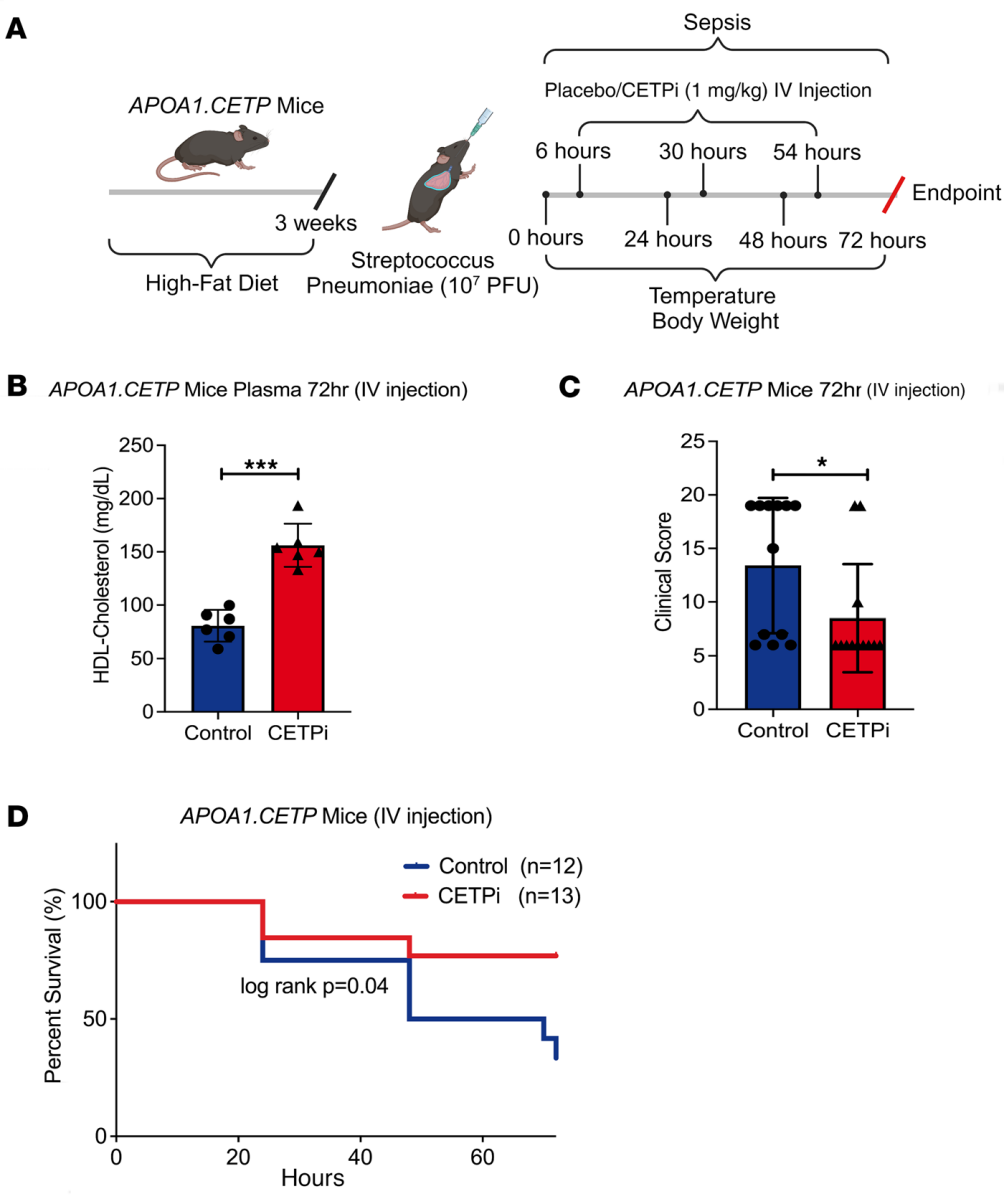
CETPi treatment after infection improves *Streptococcus pneumonia*e–induced sepsis mortality. (**A**) Schematic of the *Streptococcus pneumoniae*–induced sepsis postinfection model used in the study. (**B**) Plasma HDL-C levels in samples obtained at 0 and 72 hours after *S*. *pneumoniae* infection from female *APOA1.CETP* mice treated with control or CETPi after onset of infection (72 hours, mean ± SD, 80.8 ± 14.7 [Control, *n* = 6] versus 156.1 ± 20.1 [CETPi, *n* = 6] mg/dL, unpaired *t* test, *P* = 0.00002). (**C**) Murine Clinical Assessment Score for sepsis at 72 hours after infection for female *APOA1.CETP* mice treated after onset of infection (mean ± SD, 8.5 ± 5.0 [CETPi, *n* = 12] versus 13.4 ± 6.3 [Control, *n* = 12], unpaired *t* test, *P* = 0.046). (**D**) Kaplan-Meier plot of female *APOA1.CETP* mice survival rate (72-hour survival rate, 76.9% [CETPi, *n* = 13] versus 33.3% [Control, *n* = 12], log-rank: *P* = 0.04). Data are shown as mean ± SD. *P < 0.05, ***P < 0.001. ApoA1, apolipoprotein A-1; CETP, cholesteryl ester transfer protein; HDL, high-density lipoprotein cholesterol.

The results presented above established that pretreatment of mice with CETPi improved survival in a mouse model of *S*. *pneumoniae*–induced sepsis. To further investigate the translational potential of CETP inhibition, we tested the effect of acute CETPi treatment administered after the onset of *S*. *pneumoniae*–induced sepsis in female *APOA1.CETP* mice (Figure 6A). I.v. administration of anacetrapib after *S*. *pneumoniae* inoculation led to significantly higher plasma HDL-C concentrations at 72 hours (Figure 6B). The murine clinical assessment score was lower in CETPi-treated mice compared with control, indicating less severe sepsis (Figure 6C). Mice treated with CETPi after the onset of sepsis also preserved their body weight and temperature (Supplemental Figure 6, A and B). In concordance with these results, mice treated with CETPi had significantly increased survival compared with control (Figure 6D). These results indicate that CETPi treatment is effective at reducing the severity and mortality of pneumosepsis even when administered after the onset of the disease.

## Discussion

Here we investigated the effects and cellular mechanisms of CETP inhibition on the immune response in mouse models of *S*. *pneumoniae*–induced sepsis. The key insights from this study are that CETPi can prevent *S*. *pneumoniae*–induced dysregulated immune response and the resulting organ dysfunction by preserving HDL levels and increasing the recruitment of activated macrophages to the site of infection. CETP inhibition led to attenuation of proinflammatory cytokine release due to decreased transcription. Furthermore, we identified a previously unrecognized effect of CETP inhibition in enhancing macrophage activation and infiltration, leading to resolution of infection. Finally, we showed that CETP inhibition was effective at reducing sepsis mortality even when administered after disease onset, suggesting that CETP inhibition may represent a therapeutic approach for the treatment of sepsis.

CETP concentrations decline during severe sepsis, which may represent a physiological response to preserve the beneficial innate immune function of HDL during sepsis (33). As a result, treatment with CETPi may further augment the pool of HDL available to promote bacterial clearance. Raising HDL-C levels through CETP inhibition has failed to demonstrate a consistent benefit in reducing cardiovascular disease risk in multiple clinical trials (15). Previously, we showed that raising HDL-C levels by CETPi reduces the severity of endotoxemia and improves sepsis mortality (32). The current study provides insights into the mechanisms by which CETPi treatment leads to this beneficial effect.

The macrophage is a major phagocytic cell type, and its impaired function is a primary cause of immune paralysis, organ injury, and death in sepsis (34). In macrophages that are exposed to microbial infection, enhanced lipid synthesis has been linked to increased phagocytosis and cytokine production by providing lipids essential for maintaining the association between the actin cytoskeletal network and plasma membranes, and by the expansion of the endoplasmic reticulum and other secretory compartments to boost cytokine secretion capacity (17, 35–37). It is worth noting that CETP inhibition within monocytes may interfere with intracellular lipid metabolism, a phenomenon previously observed in adipose cells (38). This perturbation could, in turn, lead to a disproportional modification of membrane lipid constituents, ultimately culminating in monocyte activation. CETP has been proposed to be involved in trafficking cholesterol in mammals, with important implications for innate immunity (15). Inhibition of CETP results in larger HDL particles with elevated cholesteryl ester concentrations. Our findings suggest that CETP inhibition may have a dual effect on macrophage function during sepsis. Specifically, our results indicate that CETP inhibition promotes monocyte recruitment in the blood and proinflammatory macrophage recruitment into the lungs, supporting the host immune response during the early phase of sepsis. However, CETPi also appears to promote tissue repair at the late stage of sepsis, as evidenced by an increase in the percentage of *Ly6C^lo^* macrophages in the BAL.

We found that CETPi treatment led to attenuation of host inflammation, enhanced bacterial clearance, and reduced organ dysfunction and death. Our collective findings suggest that anacetrapib diminishes host inflammation through a mechanism that is dependent on the presence of HDL. Additionally, anacetrapib primes the immune system prior to bacterial exposure by activating macrophages, a process that appears to be HDL independent. Both of these mechanisms, the HDL-dependent reduction in inflammation and the HDL-independent macrophage activation, may contribute to anacetrapib’s ability to enhance survival outcomes in sepsis.

A major challenge in the treatment of sepsis caused by bacterial infection is the removal of PALs released after bacterial death in the liver (39). Overexposure to PALs can lead to inappropriate activation of the immune system (40). We found that pretreatment of *APOA1.CETP* mice with CETPi significantly enhanced monocyte activation within the blood, even prior to bacterial challenge. This preactivated state of the host immune system by CETPi is likely responsible for early bacterial recognition and phagocytosis in sepsis, leading to enhanced bacterial clearance and less extensive immune-mediated organ damage. CETPi administration before or after the onset of sepsis both blunted the decline in HDL-C during sepsis. Consequently, the relatively greater abundance of HDL in the blood plasma is likely to sequester more PALs in the blood, contributing to improved survival benefits. Importantly, CETPi worked rapidly, improving survival even when administered after pathogen exposure, suggesting CETPi as a possible treatment strategy for humans with pneumosepsis that warrants further study.

Our study has limitations that are worth mentioning. First, although we identified effects of CETP inhibition on the transcription and translation of proinflammatory cytokines and their subsequent release, the precise pharmacological mechanisms by which CETP inhibition exerts these effects remain to be determined. Second, HDL particles themselves have many immune-modulatory functions. While our in vitro results suggest that CETP inhibition enhances macrophage activation independently of HDL, the relative contributions of HDL-dependent or -independent mechanisms on improved sepsis survival in vivo remain to be determined. Similarly, our results do not distinguish between the effects of ApoA1 or other apoproteins carried by HDL on immune response. In addition, the survival in control-treated *APOA1.CETP* mice and APOE*3-Leiden.CETP mice was similar, despite differences in HDL-C levels, suggesting other factors beyond the HDL-C level that contributes to sepsis survival. Finally, it is currently unclear whether the effect of CETPi on promoting macrophage activation prior to the onset of sepsis could have detrimental effects in healthy individuals.

In summary, we show that inhibition of CETP by CETPi significantly reduces pneumosepsis mortality–enhancing macrophage activation and infiltration leading to improved bacterial clearance. These findings suggest a potential to repurpose CETPis, initially developed for the treatment of cardiovascular disease, as a therapeutic approach for sepsis.

## Methods

Supplemental Methods are available online with this article.

### Sex as a biological variable.

Our study exclusively examined female mice, which have been observed to display less variability and more robust response to CETPi therapy.

### Cell culture.

Abelson murine leukemia virus–induced macrophage cells (RAW 264.7), PBMCs, and Tohoku-Hospital-Pediatrics human acute leukemia monocyte cells (THP1) were a gift from Gordon Francis’s Lab at the University of British Columbia. Cells were cultured in Roswell-Park Memorial Institute media (RPMI-1640) with 10% FBS at 37°C in a 5% CO_2_ humidified incubator (Thermo Fisher Scientific). HDL treatment involved incubation with human HDL (catalog 361-10-0.1) at the indicated concentration or PBS control in serum-free medium (catalog 361-10).

### ELISA.

Cytokine levels in mouse plasma, BAL, tissue homogenates, RAW 264.7 cells, and THP1 cells were analyzed using commercial ELISA kits according to the manufacturer’s instructions (IL-6 mouse uncoated, 88-7064-22, Thermo Fisher Scientific; TNF-α mouse uncoated, 88-7324-22, Thermo Fisher Scientific; IL-1β mouse uncoated, 88-7013-22, Thermo Fisher Scientific; IL-1β human, BMS224-2, Thermo Fisher Scientific) according to the manufacturer’s instructions. The signals were detected using a microplate reader at the appropriate absorbance wavelength according to the manufacturer’s instructions (Molecular Devices).

### Lipid concentration assay.

HDL-C assays were performed using commercial mouse HDL Cholesterol-assay kits (79990, Crystal Chem) according to the manufacturer’s instructions. LDL-C assays were performed using commercial mouse LDL Cholesterol-assay kits (80069, Crystal Chem) according to the manufacturer’s instructions. Total cholesterol assays were performed using commercial mouse total Cholesterol-assay kits (CS0005, Sigma-Aldrich) according to the manufacturer’s instructions. Data were collected using spectraMax iD5 Microplate Reader (Molecular Devices) using an absorbance of 550–570 nm.

### Western blotting.

Cells were harvested using radio-immunoprecipitation assay buffer supplemented with protease inhibitors (04693159001, Roche). Equal amounts of protein were subjected to sodium dodecyl-sulfate polyacrylamide gel electrophoresis (SDS-PAGE) at 200 V for 1 hour and then transferred to a nitrocellulose membrane via electroblotting at 100 V for 1 hour. After blocking with Tris-buffer saline containing 0.1% Tween-20 and 5% BSA, membranes were incubated overnight with primary antibodies (1:1,000 in Tris-buffered saline with 0.1% Tween 20^®^detergent [TBST]) at 4°C, followed by horseradish peroxidase–conjugated (HRP-conjugated) secondary antibodies (1:5,000 in TBST) at room temperature for 1 hour. Enhanced chemiluminescence was used to visualize the immunoreactive bands. Primary antibodies used in this study were anti–pro–IL-1β (SC-12742; Santa Cruz Biotechnology), anti–cleaved-IL-1β (83186S, Cell Signaling Technology [CST]), anti–caspase-1 (SC-56036, Santa Cruz Biotechnology), anti–COX-2 (12282T, CST), anti-CETP (13459-1-AP, Thermo Fisher Scientific), and anti–β-actin (SC-47778, Santa Cruz Biotechnology).

### Quantitative PCR (qPCR).

Mouse tissue samples or THP1 cell lysates were harvested and isolated using commercial RNA purification kits (T201S, New England Biolabs). Total RNA concentrations in samples were determined using a Nanodrop spectrophotometer and equalized by dilution with nuclease-free water. One-step qPCR was performed using commercial kits (E3005S, New England Biolabs) according to the manufacturer’s instructions. Primers used in this study are listed in Supplemental Table 1 (Supplementary material).

### H&E staining.

Lungs and livers of mice were harvested and fixed in 10% formalin. Tissues were then embedded in paraffin and sectioned. The degree of tissue damage in experimental and control conditions was blindly reviewed and graded based on the intensity of injury and inflammation and expressed as pathological scores. A semiquantitative histopathologic scoring system of lung inflammation was developed on the basis of the presence and abundance of the perivascular/peribronchial acute inflammation (0, absent; 1, involving fewer than 20% of the perivascular/peribronchial spaces; 2, involving more than 20% but less than 40% of the perivascular/peribronchial spaces; 3, involving more than 40% but less than 60% of the perivascular/peribronchial spaces; 4, involving more than 60% but less than 80% of the perivascular/peribronchial spaces; 5, involving more than 80% of the perivascular/peribronchial spaces).

### IHC staining.

For IHC, tissue cross-sections were incubated with a primary macrophage membrane mark antibody kit (CD86 and CD206, 97624, CST) to identify specific cell types of macrophages followed by secondary antibodies using the alkaline phosphatase system to amplify signals.

### Animal studies.

Hemizygous human APOE*3-Leiden.CETP mice were bred at Leiden University Medical Center (41). Hemizygous human *APOA1*.*CETP* mice were provided by Eli Lilly laboratory (42). These transgenic mice were generated by the introduction of genomic DNA fragments that drive the expression of human *CETP* and either the dominant negative variant of common human APOE*3-Leiden or *APOA1* in the mouse liver. The mice were subjected to 2 different timelines with respect to treatment with CETPi (S7248-10MG, Selleck Chemicals) and inoculation with *S*. *pneumoniae* (33400, Cedarlane Laboratories). First, 12-week-old female APOE*3-Leiden.CETP or *APOA1.CETP* mice were fed with a semisynthetic cholesterol-enriched Western-type style diet (15% cocoa butter [w/w], 1% corn oil [w/w], 0.15% cholesterol [w/w]). After 3 weeks, the mice were continuously administered above-mentioned diet mixed with or without CETPi (10 mg/kg) for 1 week and then were administered 1 × 10^7^ colony forming unit (CFU) *S*. *pneumoniae* intratracheally to stimulate infection in the lungs. In the alternative treatment strategy, *APOA1.CETP* mice were administered *S*. *pneumoniae* (1 × 10^7^ CFU) intratracheally to induce sepsis and subsequently were administered 1 mg/kg of CETPi or saline solution control via i.v. injection at 6, 30, and 54 hours after infection. In both experiments, mice were monitored every 24 hours for body weight, temperature, and clinical assessment scores (9). Mice were humanely euthanized if core body temperature declined < 32°C or weight loss was > 20%.

### Micro-CT.

The protocol for air inflation of murine lungs is well established (43). The air inflated frozen lungs were scanned with a micro-CT scanner (HT X 225-ST, Nikon Metrology) using a cryochamber at –30°C (60 kV, 400 μA, 88 millisecond exposure, 1,800 projections over 360 degrees). The images were reconstructed at a voxel size of 40 μm in order to visualize amount if inflammation. On micro-CT, inflammation presents itself as high attenuation areas, which were visualized using a heatmap color look up table produced by the VolumeViewer tool in ImageJ (NIH).

### Flow cytometry.

BAL samples were collected from *APOA1.CETP* 4 and 72 hours after infection according to a standard protocol (44). A total of 2 mL Ca^2+^/Mg^2+^ free PBS was added to the samples and was spun down at 5,000*g* for 5 minutes. The pellet was resuspended in 1 mL Ca^2+^/Mg^2+^ free PBS. A total of 1 μL of Fixable Viability Dye eFluor 520 (65-0867-14, eBioscience, Thermo Fisher Scientific) was added to the samples and incubated at room temperature for 15 minutes. Samples were washed with 1 mL FACS staining buffer (PBS with 1% BSA and 5 mM EDTA) and blocked at room temperature for 10 minutes with TruStain FcX PLUS (156603, BioLegend). The blocked samples were then split into 100 μL aliquots and stained with a cocktail of PerCP-CD45, APC-CD11b, AF700 Ly-6G, APC/Cy7-Ly-6C, PacificBlue-I-A/I-E, PE/Cy7-SiglecF, or their respective isotype controls (PerCP-CD45 was added to the control tubes), as detailed in Supplemental Table 2 (Supplemental Table 2). A more detailed list of antibodies and controls used in this study can be found in Supplemental Table 4 (Supplementary material). Samples were stained at room temperature for 20 minutes, washed with FACS buffer, and fixed in a final concentration of 1% PFA. Stained and fixed samples were stored at 4°C until being analyzed using Gallios Flow Cytometer (Beckman Coulter). Data were analyzed using Kaluza Software v2.1 (Beckman Coulter). Compensation was performed using BD CompBeads (552844 and 55284, BD BioSciences) and adjusted using FMO and single stain controls (data not shown).

Heparinized blood samples were collected from *APOA1.CETP* mice at the baseline (0 hours) and 72 hours after infection or from APOE*3-Leiden.CETP mice at the baseline (0 hours). In total, 100 μL aliquots of blood samples were stained with a cocktail of PerCP-CD45, APC-CD11b, AF700 Ly-6G, APC/Cy7-Ly-6C, BV605-F4/80, PE/Cy7-SiglecF, PE/Cy7-NK-1.1, PE/Cy7-CD19, PE/Cy7-CD3, or their respective isotype controls (PerCP-CD45 was added to the control tubes), as detailed in Supplemental Table 3. Samples were stained at room temperature for 20 minutes and washed with FACS buffer, and RBC Lysis was performed using Beckman Coulter whole blood lysis kit (6603152, Beckman Coulter) according to the manufacturer’s recommendations. Stained samples were then fixed in a final concentration of 1% PFA. Stained and fixed samples were stored at 4°C until being analyzed using Gallios Flow Cytometer (Beckman Coulter). Data were analyzed using Kaluza Software v2.1 (Beckman Coulter). Compensation was performed using BD CompBeads (552844 and 55284, BD BioSciences) and adjusted using FMO and single-stain controls (data not shown).

### Adenovirus transfection experiments.

Raw 264.7 cells were cultured in RPMI + 10% FBS medium. At a cell density of 1 × 10^6^ raw cell count per petri dish, the cell medium was changed to RPMI without FBS. Cells were incubated with ShCETP (159650910211, Applied Biological Materials) or a scramble/nontargeting control adenovirus vector (iAAV01501, Applied Biological Materials; D-001810-01-20, Dharmacon) at the indicated multiplicity of infection (MOI). Transfected cells were harvested after 72 hours, and gene overexpression was verified with Western blotting.

### ROS activity assays.

In total, 2.5 × 10^4^ cells with indicated treatment were seeded per well in a 96-well plate the day before the experiment. Cells that reached 80% of confluency were used for ROS quantification. In total, 100 μL of 1× ROS label (ab287839, Abcam) diluted in ROS assay buffer (ab287839, Abcam) per well was added into the medium, followed by incubation at 37°C in the dark for 45 minutes. ROS label was then replaced by 100 μL of ROS assay buffer. Fluorescence at excitation/emission (Ex/Em) = 495/529 nm was measured in an end point mode.

### CETP activity assays.

CETP activity assays were performed using commercial CETP Activity-assay kits (MAK106, Sigma-Aldrich), according to the manufacturer’s instructions. Data were collected using spectraMax iD5 Microplate Reader (Molecular Devices) using a fluorometer of λ_ex_ = 465/λ_em_ = 535nm.

### Statistics.

GraphPad Prism version 9.4.0 software and ImageJ software (NIH) was used for statistical analysis. Data are displayed as means ± SD for both in vivo and in vitro derived data. Statistical analysis was performed using a 2-tailed Student’s *t* test for comparison of 2 groups. Both 1-way ANOVA and 2-way ANOVA were used for comparison of multiple groups. *P* < 0.05 was considered significant. BioRender was used to generate some of the figures with agreement number SS26EWMQNT.

### Study approval.

All animal studies were approved by the University of British Columbia animal ethics committee and performed in accordance with institutional guidelines.

### Data availability.

All data are available in the Supporting Data Values file.

## Author contributions

Conceptualization was contributed by LRB, JHB, MT, and PCNR. Methodology was contributed by LRB, PCNR, MT, and HD. Visualization was contributed by HD, WYL, LQC, and THY. Investigation was contributed by HD, WYL, LQC, and THY. Formal analysis was contributed by HD, WYL, LQC, and THY. Funding acquisition was contributed by LRB, PCNR, MT, and HD. Project administration was contributed by LRB, PCNR, and HD. Supervision was contributed by LRB. Writing of the original draft was contributed by HD, WYL, LQC, THY, and BS. Review and editing of the manuscript were contributed by LRB, JHB, PCNR, DMV, MT, KW, HD, WYL, LQC, and THY.

## Supplementary Material

Supplemental data

Supporting data values

## Version 1 04/22/2024

Electronic publication
