# Supplementary material for: CETP inhibition enhances monocyte activation and bacterial clearance and reduces streptococcus pneumonia–associated mortality in mice
Source: JCI Insight. 2024 Apr 22;9(8):e173205. doi: 10.1172/jci.insight.173205 (PMC11141867; doi:10.1172/jci.insight.173205)
Supplement: Supplemental data [file jciinsight-9-173205-s138.pdf]

SUPPLEMENTARY MATERIALS

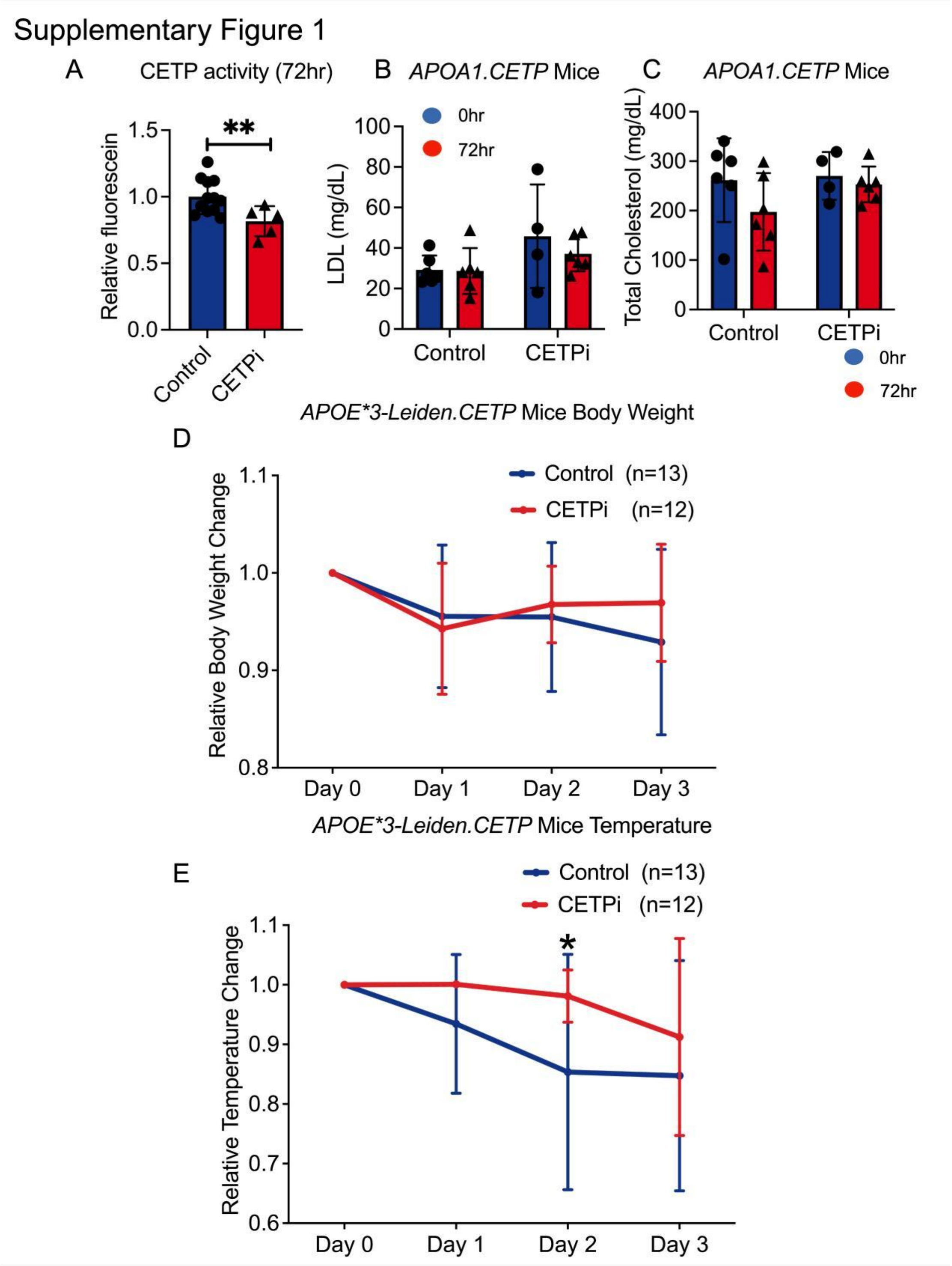

Supplementary Figure 2

A

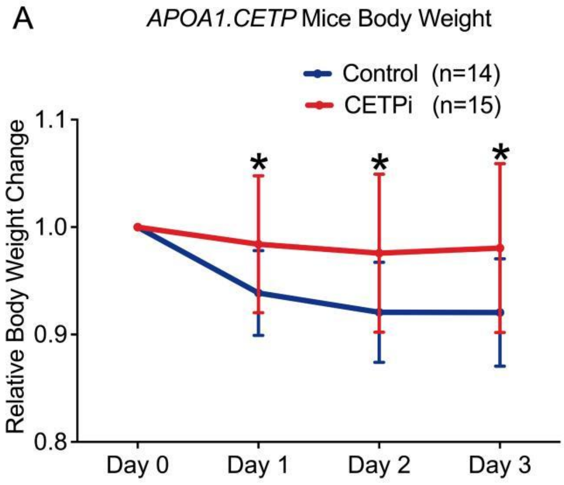

B

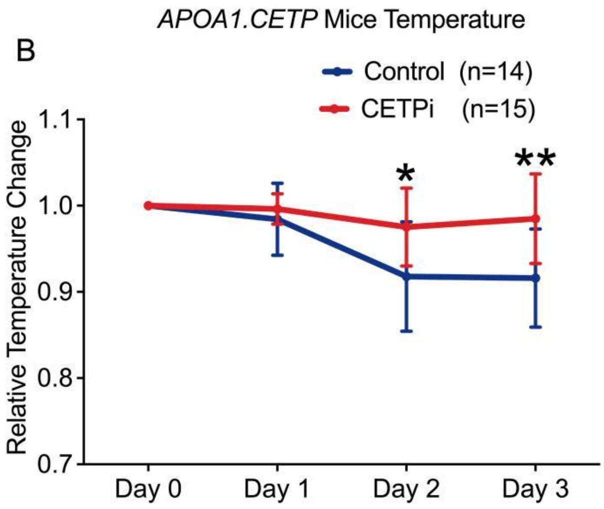

Supplementary Figure 3

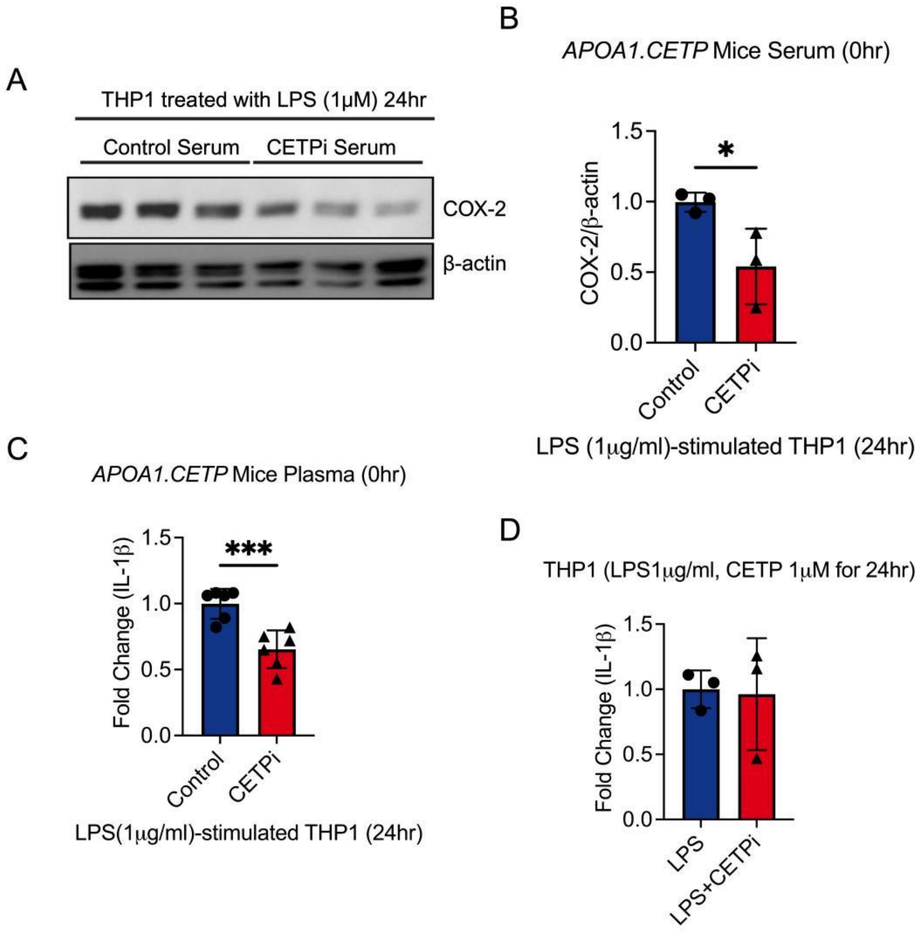

Supplementary Figure 4

A *APOE*3-Leiden.CETP* Mice

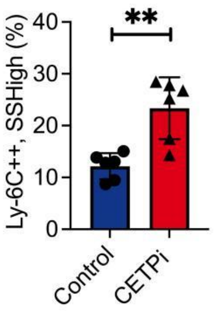

Supplementary Figure 5

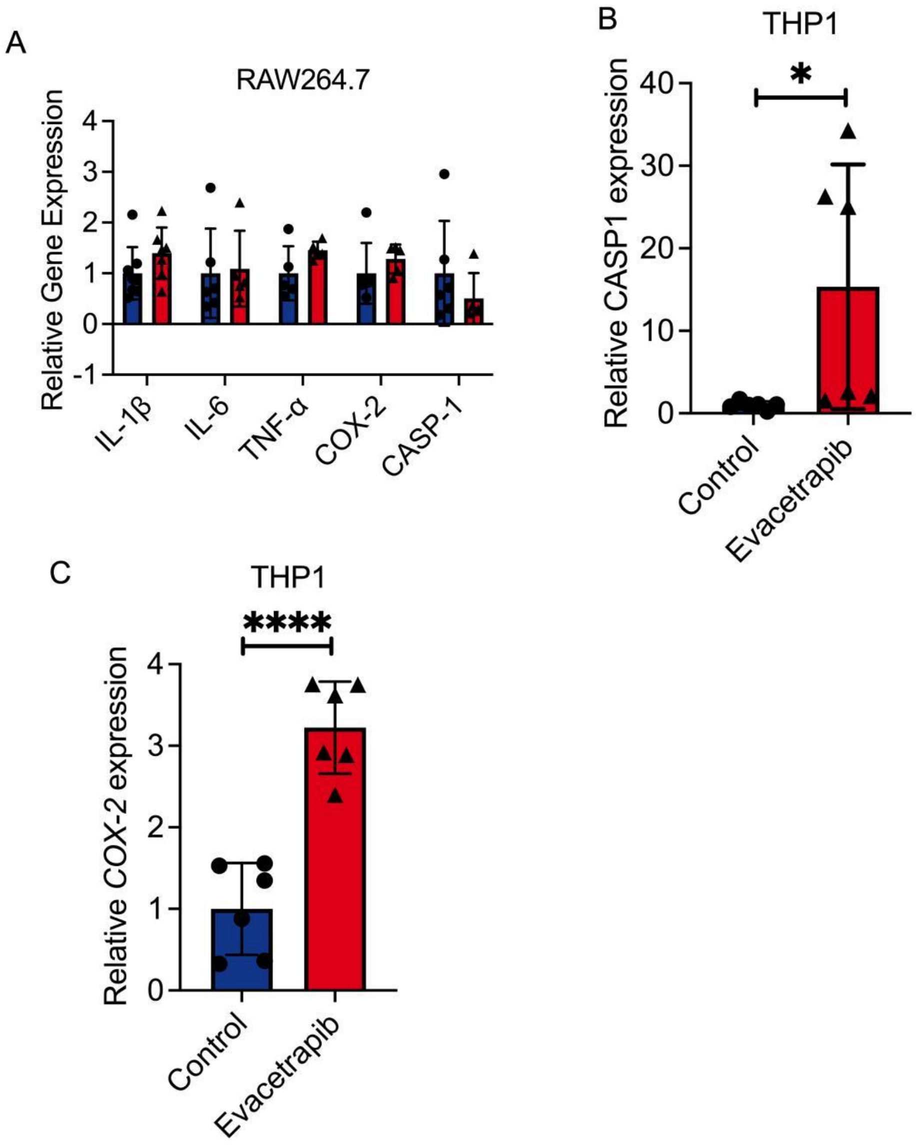

Supplementary Figure 6

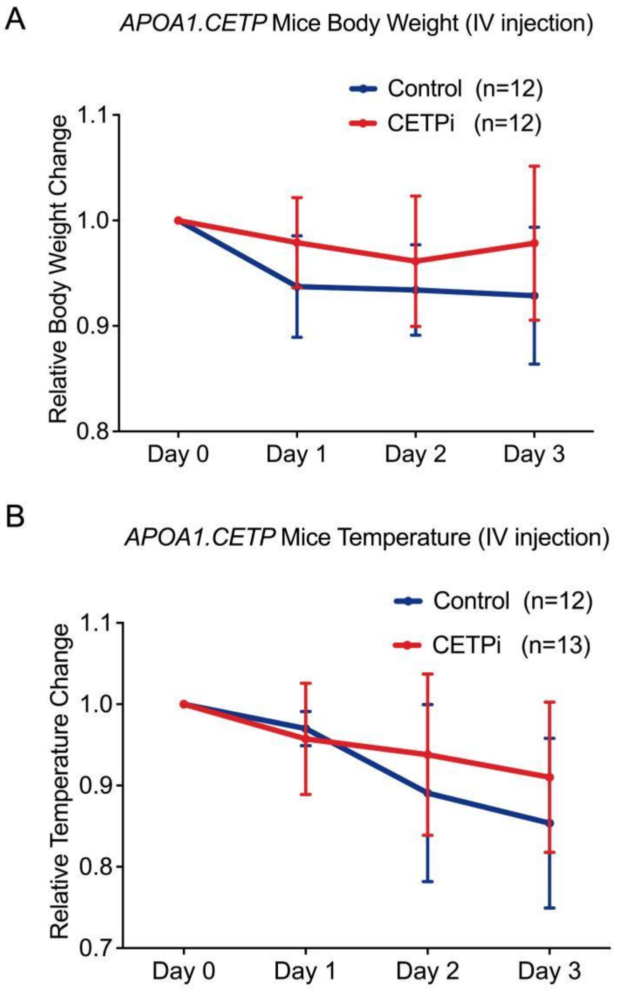

Supplementary Figure Legends:

Figure S1

A. Plasma CETP activity levels in samples obtained at 72 hours after *S. pneumoniae* infection from female *APOA1.CETP* mice treated with control or CETPi after onset of infection (72 hr, mean ± SD, 1.0±0.13 (Control, n=12) versus 0.82±0.11 (CETPi, n=5) mg/dl, unpaired *t*-test, p=0.015). **B.** Plasma LDL-C levels in samples obtained at 0 and 72 hours after *S. pneumoniae* infection from female *APOA1.CETP* mice treated with control or CETPi before onset of infection (72 hr, mean ± SD, 28.68±11.28 (Control, n=6) versus 37.12±8.54 (CETPi, n=6) mg/dl, unpaired *t*-test, p=0.17); **C.**Plasma total cholesterol levels in samples obtained at 0 and 72 hours after *S. pneumoniae* infection from female *APOA1.CETP* mice treated with control or CETPi before onset of infection (72 hr, mean ± SD, 197.47±78.37 (Control, n=6) versus 252.95±36.09 (CETPi, n=6) mg/dl, unpaired *t*-test, p=0.15). **D.** Body weight for female *APOE*3-Leiden.CETP* mice treated with placebo or anacetrapib in sepsis; **E.** Core body temperature for female *APOE*3-Leiden.CETP* mice treated with placebo or anacetrapib in sepsis. Data are presented as mean±SD, *P<0.05, **P<0.01.

Figure S2

A.Body weight for female *APOA1.CETP* mice treated with placebo or anacetrapib in sepsis. **B.** Core body temperature for female *APOA1.CETP* mice treated with placebo or anacetrapib in sepsis. Data are presented as mean±SD, *P<0.05, **P<0.01.

Figure S3

A. COX-2 expression relative to β-actin in THP1 cells treated with serum collected from either control mice or CETPi-treated mice at 0hr. LPS at indicated dose was added before serum treatment; **B.** COX-2 expression relative to β-actin in THP1 cells treated with control serum or CETPi serum (mean ± SD, 1.00±0.62 (Control, n=3) versus 0.54±0.82 (CETPi, n=3) ratio of protein expression relative to β-actin, unpaired *t*-test, p=0.046). **C.** Levels of secreted IL-1β in THP1 supernatants treated with serum collected from either control mice or CETPi-treated mice at 0hr. LPS at indicated dose was added before serum treatment. (mean±SD, 1.0±0.11 (Control, n=6) versus 0.65±0.14 (CETPi, n=6) relative protein secretion, unpaired *t*-test, p=0.0009); **D.** Levels of secreted IL-1β in THP1 supernatants treated with either DMSO or CETPi (1μM). LPS at indicated dose was added before drug treatment. (mean±SD, 1.0±0.14 (Control, n=3) versus 0.96±0.43 (CETPi, n=3) relative protein secretion, unpaired *t*-test, p=0.89)

Figure S4

A. Proportion of activated monocytes ($Ly6C^{++}SS^{High}$) in blood samples obtained at 0-hours post-infection from female *APOE*3-Leiden.CETP* mice treated with control or CETPi (mean±SD, 23.35±5.96 (CETPi, n=6) versus 12.16±2.51 (Control, n=6), unpaired *t*-test, p=0.002)

Figure S5

A. Transcriptional levels of pro-inflammatory markers in RAW 264.7 cells obtained 24 hours post-treatment with either control or CETPi (1μM) (*IL-1β*: mean±SD, 1.0±0.52(Control, n=8) versus 1.4±0.51 (CETPi, n=7) relative gene expression, unpaired *t*-test, p=0.16; *IL-6*: mean±SD, 1.0±0.88 (Control, n=6) versus 1.09±0.75 (CETPi, n=5) relative gene expression, unpaired *t*-test, p=0.86; *TNF-α*: mean±SD, 1.0±0.53 (Control, n=5) versus 1.45±0.17 (CETPi, n=5) relative gene expression , unpaired *t*-test, p=0.10; *COX-2*: mean±SD, 1.0±0.6 (Control, n=6) versus 1.28±0.28 (CETPi, n=5) relative gene expression, unpaired *t*-test, p=0.36; *CASP-1*: mean ± SD, 1.0±1.03 (Control, n=6) versus 0.5±0.5 (CETPi, n=5) relative gene expression, unpaired *t*-test, p=0.35); **B.** Transcriptional levels of pro-inflammatory markers in THP1 cells obtained 24 hours post-treatment with either DMSO or evacetrapib (2μM) (*CASP-1*: mean±SD, 1.0±0.48(Control, n=6) versus 15.33±14.82 (CETPi, n=6) relative gene expression, unpaired *t*-test, p=0.04; **C.** *COX-2*: mean±SD, 1.0±0.56 (Control, n=6) versus 3.22±0.56 (CETPi, n=6) relative gene expression, unpaired *t*-test, p=0.00004;

Figure S6

A.Body weight for female *APOA1.CETP* mice treated after onset of infection in sepsis. **B.** Core body temperature for female *APOA1.CETP* mice treated after onset of infection in sepsis.

Supplementary Tables:

Table 1: Primers for Pro-inflammatory Cytokines

Target	Forward Primer	Reverse Primer
**ACTB (IDT, USA)**	CATTGCTGACAGGATGCAGAAGG	TGCTGGAAGGTGGACAGTGAGG
**CCL-5 (IDT, USA)**	GCTGCTTTGCCTACCTCTCC	TCGAGTGACAAACACGACTGC
**CXCL-10 (IDT, USA)**	GCTGGGATTCACCTCAAGAA	CTTGGGGACACCTTTTAGCA
**IL-6 (IDT, USA)**	ACAACCACGGCCTTCCCTAC	TCTCATTTCCACGATTTCCCAG
**IL-1β (IDT, USA)**	GCCTCGTGCTGTCGGACCCA	TGAGGCCCAAGGCCACAGGT

**lytA (IDT, USA)**	ACGCAATCTAGCAGATGAAGCA	TCGTGCGTTTTAATTCCAGCT
**TNF-α (IDT, USA)**	GTCCCCAAAGGGATGAGAAGTT	GTTTGCTACGAGGTGGGCTACA
**COX-2 (murine) (IDT, USA)**	GCGACATACTCAAGCAGGAGCA	AGTGGTAACCGCTCAGGTGTTG
**CASP-1 (murine) (IDT, USA)**	GGCACATTTCCAGGACTGACTG	GCAAGACGTGTACGAGTGGTTG
**COX-2 (human) (IDT, USA)**	CGGTGAAACTCTGGCTAGACAG	GCAAACCGTAGATGCTCAGGGA
**CASP-1 (human) (IDT, USA)**	GCTGAGGTTGACATCACAGGCA	TGCTGTCAGAGGTCTTGTGCTC

Table 2. List of antibodies used for flow cytometric analysis of BAL samples from APOA1.CETP mice

**Antibody**	**Tag**	**Clone**	**Input***
**CD45**	PerCP	30-F11	0.25 µg
**CD64 (FcγRI)**	PE	X54-5/7.1	1 µg
**CD11b**	APC	M1/70	0.25 µg
**I-A/I-E (MHCII)**	Pacific Blue	M5/114.15.2	0.25 µg

**Ly-6G**	AF700	1A8	0.25 µg
**Ly-6C**	APC/Cy7	HK1.4	0.25 µg
**Siglec F (CD170)**	PE/Cy7	S17007L	0.25 µg

***per 1x10^6 cells in 100 µL**

Table 3. List of antibodies used for flow cytometric analysis of blood samples from APOA1.CETP mice

**Antibody**	**Tag**	**Clone**	**Input***
**CD45**	PerCP	30-F11	0.25 µg
**CD11b**	APC	M1/70	0.25 µg
**F4/80**	BV 605	T45-2342	0.25 µg
**Ly-6G**	AF700	1A8	0.25 µg
**Ly-6C**	APC/Cy7	HK1.4	0.25 µg
**Dump Channel:**			
**CD19**	PE/Cy7	6D5	0.25 µg
**CD3**	PE/Cy7	17A2	0.25 µg
**NK-1.1**	PE/Cy7	S17016D	0.5 µg
**Siglec F (CD170)**	PE/Cy7	S17007L	0.25 µg

***per 100 µL blood**

Table 4. Detailed list of antibodies and corresponding IgG controls used for flow cytometric analysis of BAL and blood samples from APOA1.CETP mice:

**Supplier**	**Cat #**	**Description**	**Clone**	**Input***	**Panels:**
**Biolegend**	103129	PerCP anti-mouse CD45	30-F11	0.25 µg	BAL, Blood
**Biolegend**	101211	APC anti-mouse/human	M1/70	0.25 µg	BAL, Blood

		CD11b			
**Biolegend**	400611	APC Rat IgG2b, κ Isotype Ctrl	RTK4530	0.25 μg	BAL, Blood
**Biolegend**	127621	Alexa Fluor 700 anti-mouse Ly-6G	1A8	0.25 μg	BAL, Blood
**Biolegend**	400528	Alexa Fluor 700 Rat IgG2a, κ Isotype Ctrl	RTK2758	0.25 μg	BAL, Blood
**Biolegend**	128025	APC/Cy7 anti-mouse Ly-6C	HK1.4	0.25 μg	BAL, Blood
**Biolegend**	400719	APC/Cy7 Rat IgG2c, κ Isotype Ctrl	RTK4174	0.25 μg	BAL, Blood
**Biolegend**	155527	PE/Cy7 anti-mouse CD170 (Siglec-F)	S17007L	0.25 μg	BAL, Blood
**Biolegend**	400521	PE/Cy7 Rat IgG2a, κ Isotype Ctrl	RTK2758	0.25 μg	BAL, Blood
**Biolegend**	139304	PE anti-mouse CD64 (FcγRI)	X54-5/7.1	1 μg	BAL
**Biolegend**	400112	PE Mouse IgG1, κ Isotype Ctrl	MOPC-21	1 μg	BAL
**Biolegend**	107619	Pacific Blue anti-mouse I-A/I-E	M5/114.15.2	0.25 μg	BAL
**Biolegend**	400627	Pacific Blue Rat IgG2b, κ Isotype Ctrl	RTK4530	0.25 μg	BAL
**Biolegend**	156513	PE/Cy7 anti-mouse NK-1.1	S17016D	0.5 μg	Blood

**Biolegend**	400253	PE/Cy7 Mouse IgG2a, κ Isotype Ctrl	MOPC-173	0.5 µg	Blood
**Biolegend**	115519	PE/Cy7 anti-mouse CD19	6D5	0.25 µg	Blood
**Biolegend**	400522	PE/Cy7 Rat IgG2a, κ Isotype Ctrl	RTK2758	0.25 µg	Blood
**Biolegend**	100219	PE/Cy7 anti-mouse CD3	17A2	0.25 µg	Blood
**Biolegend**	400617	PE/Cy7 Rat IgG2b, κ Isotype Ctrl	RTK4530	0.25 µg	Blood
**Biolegend**	400655	Brilliant Violet 421 Rat IgG2b, κ Isotype Ctrl	RTK4530	0.5 µg	Blood
**BD Biosciences**	743281	Brilliant Violet 605 anti-mouse F4/80	T45-2342	0.25 µg	Blood
**BD Biosciences**	563144	Brilliant Violet 605 Rat IgG2a, κ Isotype Ctrl	R35-95	0.25 µg	Blood

***(per ~1x10^{6} cells in 100 µL, or per 100 µL of blood)**

<u>Supplementary Methods:</u>

Flow cytometry

BAL: the samples were first gated on forward scatter (FSC) and side scatter (SSC) to remove most of the debris and contaminating RBCs. Cell aggregates were then removed using a FSC width gate.CD45 was used to identify leukocytes that were then gated for viability using the Fixable Viability Dye eFluor™ 520. To have the purest viable leukocyte gate, cells gated loosely for higher fluorescence in CD45 were fed into Viability vs FSC, then Viability vs SSC and then fed back into a Viability vs CD45 graph. This multi-level gating allowed us to not only have a pure gate but also

check for any leukocytes with lower CD45 expression as well as cells with higher autofluorescence in CD45 channel. Macrophages were identified as $CD45^{+}$ $CD64^{+/low}$ Ly-$6G^{-}$ and then subdivided into Tissue Resident Alveolar Macrophages (TR-AM) that are $SiglecF^{+}$, Infiltrating Monocyte derived (monocytic) Alveolar Macrophages (Mo-AM) that are $CD11b^{+}$. Interstitial Macrophages (IM), that are also $CD11b^{+}$ are not naturally found in BAL. MHCII expression was used to exclude that there is no contaminating IMs in Mo-AM gate. Likewise, the presence of Dendridic Cells (DC) that may also end up in the BAL were checked using CD64 and MHCII expression. After Alveolar Macrophages were sub-phenotyped; they were analyzed for their Ly-6C and MHCII fluorescence (normalized to IgG, gated based on SSC, FSC, CD45 and viability). Blood: FSC and SSC were used to gate the debris and RBCs out, FSC width was used to gate out the cell aggregates and then CD45 was used to obtain a clean population of leukocytes. A dump channel was used to gate out Eosinophils (SiglecF), B cells (CD19), T cells (CD4) and NK cells (NK1.1). $CD11b^{+}$ leukocytes were selected, and monocytes and neutrophils were gated as $CD45^{+}$ $CD11b^{+}$ $Dump^{-}$ Ly-$6G^{-}$ and $CD45^{+}$ $CD11b^{+}$ $Dump^{+/-}$ Ly-$6G^{+}$ respectively. Monocytes were then investigated for their expression of Ly-6C (inflammatory marker), CCR2 (found on young monocytes leaving the bone marrow and necessary for recruitment to the site of inflammation) and F4/80 (maturation marker).
